# Exposure to Sexually Explicit Materials and Feelings after Exposure among Adolescents in Nine European Countries: The Role of Individual Factors and Social Characteristics

**DOI:** 10.1007/s10508-022-02401-9

**Published:** 2022-08-29

**Authors:** Michaela Lebedíková, Vojtěch Mýlek, Kaveri Subrahmanyam, David Šmahel

**Affiliations:** 1 Interdisciplinary Research Team On Internet and Society, Faculty of Social Studies, Masaryk University, Jostova 10, Brno, Czechia; 2 College of Natural and Social Sciences, Department of Psychology, California State University Los Angeles, Los Angeles, CA USA; 3 Department of Machine Learning and Data Processing, Faculty of Informatics, Masaryk University, Brno, Czechia

**Keywords:** Adolescents, Sexually explicit material, Parental mediation, Cross-national sampling

## Abstract

Research on adolescents’ sexual exposure has mostly focused on negcative outcomes using a risk-based lens, and there is little work on the factors that may predict exposure, as well as youths’ emotional responses to sexual content. Using a cross-national sample, the present study examined the associations of individual (sensation seeking and emotional problems) and social characteristics (the quality of family environment, including active and restrictive parental mediation) with adolescents’ exposure to sexually explicit materials and their feelings after exposure. The survey included 8,820 11- to 16-year-olds (M_age_ = 13.36 years, SD = 1.62, 48.0% male) from nine European countries (Czech Republic, Finland, Malta, Poland, Portugal, Romania, Serbia, Spain, Switzerland). The results revealed that although there were differences in the prevalence of youths’ sexual exposure by country, there were also similarities in the characteristics underlying exposure and subsequent feelings across different country contexts. No significant relationship was found between active parental mediation and exposure in most countries, and the findings regarding restrictive parental mediation were mixed. Although the majority of the participants reported neutral feelings, there were gender differences in feeling happy and upset after exposure. Overall, the results suggest that exposure may not be as distressing to youth as prevalent risk-focused narratives have suggested.

**Supplementary Information:**

The online version contains supplementary material available at 10.1007/s10508-022-02401-9.

## Introduction

Sexually explicit materials have become easily and anonymously accessible within offline and online contexts for both adolescents and adults (Alexandraki et al., 2018). Extant research on youth has mostly focused on the negative correlates of exposure to sexually explicit materials (e.g., Alexandraki et al., 2018; Kohut & Štulhofer, 2018; Mattebo et al., 2018; Peter & Valkenburg, 2011; Štulhofer et al., 2019) and a few studies have focused on the positive correlates (e.g., Litsou et al., 2020; Ševčíková & Daneback, 2014). Previous research has revealed that various individual (e.g., sensation seeking and emotional problems) and social characteristics (e.g., quality of the family environment) play an important role in predicting exposure to sexually explicit materials (Beyens et al., 2015; Peter & Valkenburg, 2006; Ševčíková et al., 2014; Vandenbosch, 2015; Vandenbosch & Peter, 2016; Weber et al., 2012).

With regard to the role of the family, there is limited research on the effectiveness of parental efforts to manage their adolescents’ exposure to online sexually explicit material. Another issue is youths’ emotional reactions to exposure (Cameron et al., 2005; Doornwaard et al., 2017; Lewis, 2018; Martelozzo et al., 2020, Smith, 2013), and no study to date has investigated the individual and social characteristics that are associated with positive and negative feelings after exposure to sexually explicit materials. Finally, extant research has mostly been conducted on national samples (i.e., Doornwaard et al., 2015; Martellozzo et al., 2020; Tomić et al., 2018; Vandenbosch & Peter, 2016) and few studies have used a cross-national perspective. To better understand the consequences of adolescents’ exposure to sexually explicit materials, it is crucial to explore the correlates of positive and negative feelings after exposure. Such knowledge can help identify individuals who may benefit from interventions designed to prevent the harmful effects of exposure. The current study explores the following research questions:

### RQ1:

What is the relationship between individual and social characteristics (including active and restrictive parental mediation) and exposure to sexually explicit materials in nine European countries (Czech Republic, Finland, Malta, Poland, Portugal, Romania, Serbia, Spain, Switzerland)?

### RQ2:

What is the relationship between individual and social characteristics and adolescents’ feelings after exposure to sexually explicit materials?

### Adolescents’ Media Use and Well-being

Overall, European adolescents mostly access the internet through their mobile phones (ranging from 70 to 89% across sixteen countries), and in eleven countries, more than 80% of them did so daily (Smahel et al., 2020). They report that they spend 134 to 219 min per day online; in the past ten years, this number has doubled in many European countries, suggesting a similar trend across Europe (Smahel et al., 2020). Research has also shown similarities in European adolescents’ consumption and use of cinema and film (Soto-Sanfiel et al., 2019).

**Fig. 1:**
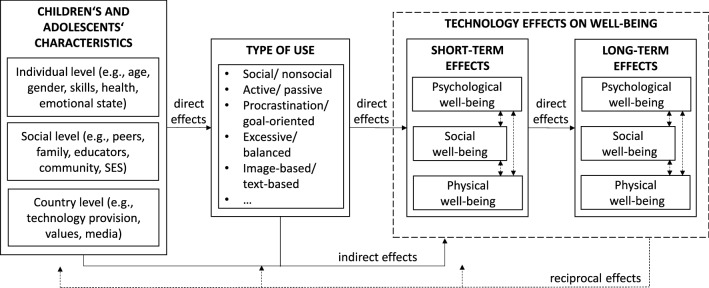
Conceptual model of information and communication technology effects on well-being (Dědková et al., 2022)

The internet has become an important avenue for adolescents to explore their sexuality (Scott et al., 2020). Building social, emotional, and cognitive skills connected to sexuality is a major developmental task during adolescence; one that presents risks and opportunities (Arbeit, 2014; Havighurst, 1948). The theoretical foundation for this study draws on Arbeit’s skills-based model of adolescent sexual development (Arbeit, 2014), which posits that adolescents can promote positive potential in their own sexuality while managing risk in three areas—sexual selfhood, sexual empowerment, and sexual negotiation. In all three areas, media—including sexually explicit materials, defined in this study as obviously sexual pictures, photographs or videos showing people naked or having sex, including pornography—play a prominent role in shaping the messages that youth receive (Arbeit, 2014; Collins et al., 2017), and thus influence their development as well as their short- (e.g., immediate reactions, Valkenburg & Peter, 2013) and long-term well-being (e. g., mental health, Dědková et al., 2022). At this point in time, adolescents mostly access sexually explicit materials online, where access is easier and more anonymous compared to offline settings (Alexandraki et al., 2018; Šmahel et al., 2020); hence, a useful framework for understanding these media effects on adolescents’ well-being is the model of information and communication technologies’ effect on well-being (Dědková et al., 2022, see Fig. 1). This model extends the Differential Susceptibility to Media Effects Model (DSMM), which explains the conditionality of media effects (Valkenburg & Peter, 2013) in terms of differential susceptibility variables that are reorganized and expanded at the individual, social, and country levels; outcomes are conceptualized in terms of short- and long-term well-being. Similar to the DSMM, the information and communication technologies model defines children’s and adolescents’ characteristics, at the individual, social, and country levels. According to the model, these characteristics have a direct effect on media use, which then directly affects both short- and long-term well-being. Additionally, the state of users’ well-being has a reciprocal effect on their characteristics and media use.

**Fig. 2:**
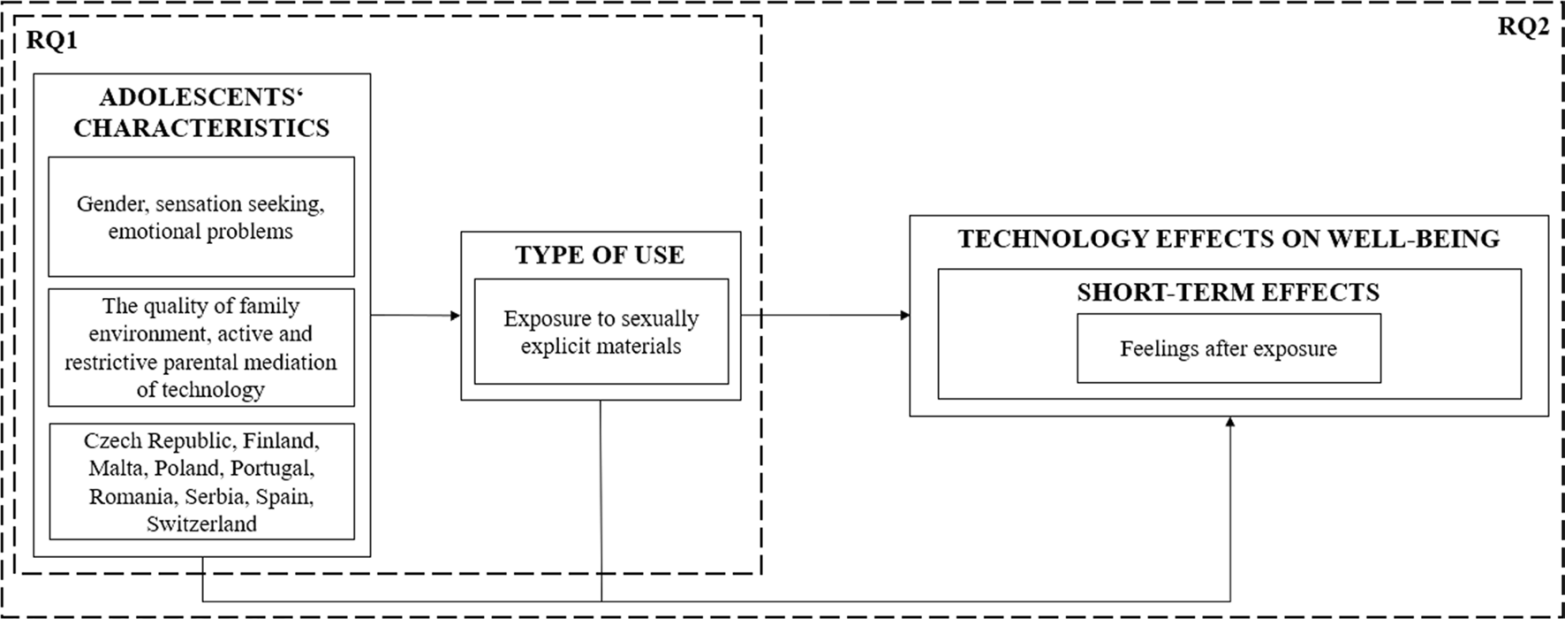
Logic of this study based on the model by Dědková et al., 2022

The logic for our analyses is adapted from the model in Fig. 2. Our first analysis related to RQ1 examined the relationship between individual (gender, sensation seeking, emotional problems), social (the quality of family environment, active and restrictive parental mediation of technology), and country (Czech Republic, Finland, Malta, Poland, Portugal, Romania, Serbia, Spain, Switzerland) characteristics and a specific type of media content use—exposure to sexually explicit materials. The second analysis related to RQ2 investigated youths’ feelings after exposure (short-term well-being) as well as the individual (gender, sensation seeking, emotional problems), social (the quality of family environment), and country (Czech Republic, Finland, Malta, Poland, Portugal, Romania, Serbia, Spain, Switzerland) characteristics associated with it.

### Adolescents’ Exposure to Sexually Explicit Materials

As adolescents explore their sexuality, it is normative for them to seek out sexually explicit materials offline (Steinberg et al., 2019) as well as online (Subrahmanyam & Šmahel, 2011). Across Europe, there is variability in adolescents’ reported exposure to sexually explicit materials as a function of both country and gender (Andrie et al., 2021; Donevan et al., 2022; Ševčíková & Daneback, 2014; Šmahel et al., 2020). Their reasons for seeking sexually explicit materials are similarly varied and include wanting to be sexually aroused, satisfying curiosity about sex, being in a romantic relationship, wanting to learn more about it, and for some, a type of friendly socialization (Löfgren-Mårtenson & Månsson, 2010; Ševčíková & Daneback, 2014; Smith, 2013).

### Individual Characteristics Associated with Exposure to Sexually Explicit Materials

Gender differences in adolescents’ exposure to sexually explicit materials have been consistently found across European countries (Šmahel et al., 2020). Boys differ significantly from girls in their reasons for exposure to sexually explicit materials, (compared to girls, boys more often seek sexual content out of curiosity and arousal), as well as intentionality and frequency of consumption (Beyens et al., 2015; Doornwaard et al., 2015; Milas et al., 2019; Ševčíková & Daneback, 2014; Vandenbosch & Peter, 2016). Researchers have attributed these gender differences to prevalent gender stereotypes (as girls may be less willing to disclose that they consume sexually explicit materials) and to different developmental trajectories; for example, boys report earlier age at debut of masturbation compared to girls (Ševčíková & Daneback, 2014).

Research suggests that two characteristics related to adolescents’ exposure to sexually explicit material are sensation seeking (Beyens et al., 2015; Peter & Valkenburg, 2006; Ševčíková et al., 2014) and emotional problems (Doornward et al. 2016, Mattebo et al., 2018; Štulhofer et al., 2019). Sensation seeking is ‘the need for varied, novel, and complex sensations and experiences and the willingness to take physical and social risks for the sake of such experiences’ (Zuckerman, 1994, p. 27). The content of sexually explicit materials may make it compelling to high sensations seekers; adolescents who scored high in sensation seeking were more strongly involved in sexual activities (Savin-Williams & Diamond, 2004) and tended to search more often for sexual content (Collins et al., 2004). Whereas longitudinal research has revealed the important antecedent role of sensation seeking in exposure to sexually explicit materials (Koletić, 2017), one study has found no relation between the two (Vandenbosch & Peter, 2016).

Emotional problems, denoting symptoms of depression and anxiety, are another psychological characteristic that has been investigated as a predictor of exposure to sexually explicit materials. Consuming pornography may be a way of coping with feelings of discomfort and emotional stress for some adolescents (Mattebo et al., 2018, p. 237–238), and may also be associated with poor mental health (Tsitsika et al., 2009; Ybarra & Mitchell, 2005). At the same time, other research has not found an association between mental health and pornography consumption (Mattebo et al., 2018; Štulhofer et al., 2019). Given that the research has yielded both positive and negative associations between emotional problems and exposure, it is important to further examine the potential association between them.

### Social Characteristics Important for Exposure to Sexually Explicit Materials

Previous studies have also suggested that a good quality family environment (i.e., safety, absence of conflicts, support of the children, and communication) may be an important factor that protects adolescents from the negative effects of many risky behaviors, including exposure to sexually explicit materials (Ma et al., 2017b; Shek & Ma, 2012). Other studies have found similar support for related characteristics, such as emotional bonding with a caregiver (Ybarra & Mitchell, 2005) and family commitment (Mesch, 2009). While different conceptualizations of family variables make it difficult to see what aspects are the most important in lowering exposure, the results suggest that a positive family environment may protect adolescents from exposure to sexually explicit materials.

Another potentially important family variable that may influence youths’ exposure to sexually explicit materials is parental mediation of technology use, which refers to the strategies that parents adopt to manage their children’s use of the internet. This is important to consider given that sexual materials are mostly accessed on devices connected to the internet (Šmahel et al., 2020). Two types of parental mediation of technology use have been described: restrictive and enabling (Livingstone et al., 2017a, 2017b). Restrictive parental mediation of technology use aims to limit children’s internet use, for example, by introducing time limits for using the computer or the internet, as well as bans and rules concerning specific content. Enabling parental mediation involves explaining and talking to children about the risks and benefits of the internet. Such conversations are aimed to encourage them to use the internet in a positive way and encompasses a range of behaviors, such as active mediation of internet use and internet safety, technical controls (i.e., filtering of content), and parental monitoring (i.e., checking browser history). Whereas restrictive parental mediation may be more effective in preventing online risks, it also precludes youth from taking advantage of online opportunities. Enabling parental mediation is believed to allow youth to explore the opportunities, as well as risks of technologies (Livingstone et al., 2017a, 2017b).

Extant research on parental mediation regarding sexually explicit materials consists of two areas: general parental mediation of internet or media use (such as Nikken & de Graaf, 2013; Ševčíková et al., 2014) and specific parental mediation that also includes talking about sex and sexuality-related to media with youth (Nikken & de Graaf, 2013; Boniel-Nissim et al., 2019). In Europe, restrictive parental mediation of technology was negatively associated with exposure (Ševčíková et al., 2014); however, related research shows that restricting media use may backfire or have no effect (Boniel-Nissim et al., 2019; Nikken & de Graaf, 2013). Regarding active parental mediation of technology use, Ševčíková et al. (2014) found no significant association with sexual exposure and Nikken and de Graaf (2013) found that neither active mediation of sex-related content in media nor discussing sexuality-related issues with parents influenced sexual experience in both boys and girls. As parents mediate many risks associated with internet use (e.g., content or excessive use risks), they may employ a more general approach to the mediation of technology use (Symons et al., 2017), and thus, it is important to investigate whether such a broad strategy is effective in decreasing the risk of exposure to sexually explicit materials.

### Role of Other Variables in Exposure to Sexually Explicit Materials

Research has also revealed the role of other predictors such as age and media use in adolescents’ exposure to sexually explicit materials. Studies across the globe have consistently demonstrated that older youth report greater exposure to sexually explicit materials (Ševčíková et al., 2014; Perry & Heyward, 2017; Ma et al., 2017a). Additionally, there is a positive association between the time spent online and exposure to sexually explicit materials among boys (Mattebo et al., 2013), and in both genders (Luder et al., 2011; Ševčíková et al., 2014).

### Feelings after Exposure to Sexually Explicit Materials

Sexually explicit materials have often been framed as threatening to adolescents’ well-being and development (Albury, 2014), and research has mostly investigated the issue with a negative lens (Koletić, 2017). Differentiation between risk and harm is crucial: The occurrence of an event that is risky in its potential consequences does not mean that harm or ‘actual physical or mental damage’ will subsequently also occur (Livingstone & Görzig, 2014). The consumption of offline and online sexually explicit materials has become a normal part of sexual development among youth (Steinberg et al., 2019; Subrahmanyam & Smahel, 2011), with some evidence of positive effects (Litsou et al., 2020; Löfgren-Mårtenson & Månsson, 2010; Ševčíková & Daneback, 2014; Smith, 2013). Research on other aspects of the issue is still scarce (Peter & Valkenburg, 2016), and adolescents’ reactions to sexually explicit materials have not received as much attention as questions about the behavioral and attitudinal effects of exposure to this content. It is important to better understand adolescents’ reactions when exposed to sexually explicit materials in order to assess whether exposure to sexually explicit materials affects their well-being and to identify groups that may be most at risk for harm. Research to date on adolescents’ experiences and feelings associated with exposure is mostly qualitative (Cameron et al., 2005; Doornwaard et al., 2017; Lewis, 2018; Smith, 2013), except for one recent quantitative study (Martellozzo et al., 2020).

Qualitative studies show the gendered nature of adolescents’ reactions to sexual content; whereas girls were exposed less frequently than boys, they reported reacting more negatively to it, possibly because they were more sensitive to sexual content (Cameron et al., 2005; Doornward et al., 2017). In a Dutch study, boys consumed sexually explicit materials more frequently than girls and tended to say that it felt arousing (Doornwaard et al., 2017). Gender differences have also been found in what is viewed as sexually explicit (Lewis et al., 2018); girls considered women in sexual, provocative, or suggestive poses’ to be sexually explicit, whereas boys considered ‘full nudity’ to be so. Although qualitative studies suggest there may be differences in reaction by gender, there has been no investigation of adolescents’ feelings (positive and negative) and its covariates after exposure to sexually explicit materials.

### The Importance of Individual and Social Variables in Youths’ Feelings After Exposure to Sexually Explicit Materials

Research has also found that negative feelings after exposure may decrease with time after a first viewing (Martellozo et al., 2020), possibly because youth may become desensitized to sexually explicit content as they age. In addition to the foregoing factors, youths’ feelings after exposure to sexually explicit materials may be affected by a range of different individual and social characteristics. However, no previous studies have identified the characteristics of adolescents who may experience positive or negative feelings after exposure to sexually explicit materials. Previous research on exposure to sexually explicit materials and online risks has suggested that the following characteristics may relate to feelings after exposure to sexually explicit materials: Adolescents who scored high on sensation seeking tend to be more interested in risky and explicit content (Livingstone & Görzig, 2014). On the other hand, those who scored lower on sensation seeking ‘may be more easily upset when they encounter them’ (ibid.). Although adolescents with emotional problems may be more likely to seek risky content as a means of coping, Livingstone and Görzig (2014) suggested that their poorer mental health may also make them more vulnerable to experiencing harm. Good quality of the family environment may be a protective factor, not only from risk but also from potential negative effects when engaging in risky behaviors (Tomić et al., 2018), including experiencing harm (negative feelings) from exposure. We did not find any study describing associations between parental mediation and feelings after exposure to sexually explicit materials.

### The Current Study

Adolescents’ exposure to sexually explicit materials has been studied in association with several individual and family characteristics, but prior research has not systematically investigated the role of parental mediation in such exposure. Further, the limited research on adolescents’ feelings after exposure to sexually explicit materials is mostly qualitative, and no study to date has investigated the individual characteristics that may be associated with youths’ feeling happy or upset after exposure to sexually explicit content. To investigate these questions, we analyzed survey data from 11- to 16-year-olds in the EU Kids Online Project with the aim of exploring the associations of individual and family characteristics and exposure to sexually explicit materials and adolescents’ feelings (happy or upset) after exposure.

In our first analysis related to RQ1, we explored the associations of gender, sensation seeking, emotional problems, and quality of the family environment with exposure to sexually explicit materials when controlling for age and time spent online. We included active and restrictive parental mediation in one model with both individual and family characteristics. In our second analysis related to RQ2, we examined the associations of gender, sensation seeking, emotional problems, and quality of the family environment with feeling happy and upset after exposure to sexually explicit materials while controlling for time spent online and age. Finally, although prior work on youths’ exposure to sexually explicit online content has been conducted in several different countries and cultural contexts, this is one of the first studies to systematically investigate these associations in a sample of nine European countries (Czech Republic, Finland, Malta, Poland, Portugal, Romania, Serbia, Spain, Switzerland). By conducting cross-national comparisons of the results, the study explores whether there are similarities versus differences in the factors underlying youths’ exposure to sexually explicit material across different country contexts.

## Method

In the current study, we used existing data from the EU Kids Online Project which surveyed nationally representative samples of adolescents in nineteen European countries about their various online experiences. From the nineteen countries, we selected only those meeting the following criteria: (1) their data included questions about adolescents’ exposure to sexually explicit materials and their feelings afterwards (in some countries, the survey did not include these latter questions); (2) the data were collected from adolescents ranging in age from 11 to 16 years (some countries focused on different age ranges, e.g., 12–17 in Germany); and (3) the data were collected at schools and not at home. The location of data collection is important because in home-based surveys, adolescents have reported lower rates of exposure to sexually explicit materials (Šmahel et al., 2020), suggesting less willingness to disclose this online activity in the vicinity of their parents. Data from nine countries satisfied the above selection criteria—Czechia, Finland, Malta, Poland, Portugal, Romania, Serbia, Spain, and Switzerland.

Sampling and data collection were done at general, vocational, regular, and academic schools using proportional stratified random-clustered sampling (based on age, gender, region, and if applicable, urban/rural status in order to provide data representative of the targeted population). Data were collected between October 2017 and April 2019 using computer-assisted personal interviewing/computer-aided web interviewing (CASI/CAWI) in Czechia, Finland, Poland, Portugal, Romania and pen-and-paper personal interviews (PAPI) in Malta, Serbia, Spain, and Switzerland. Questionnaires were administered in schools by trained administrators. The data collection was approved by relevant ethics bodies in each country. Prior to data collection, assent from participants and informed consent from their parents were obtained. The data were anonymized after collection. For detailed information about the ethical standards and other aspects of the data collection, see (Zlamal et al., 2020).

### Participants

Across the nine included countries, a total of 10,401 adolescents aged 11–16 were surveyed. However, 1,581 adolescents had missing values in questions about their exposure to sexually explicit materials (i.e., responded ‘*I don’t know*,*’* ‘*Prefer not to answer*,*’* or skipped the question; see Table 1) and were not included in the analyses. Thus, our final sample was comprised of 8,820 adolescents aged 11–16 (M_age_ = 13.36, SD = 1.62, 48.0% boys). Excluded participants did not differ from included ones with regard to their age (*t*(10,399) = 1.45, *p* = 0.148), time spent online (*t*(9,547) = 1.54, p = 0.124), sensation seeking (*t*(1,863) = 0.22, *p* = 0.830), or emotional problems (*t*(9,971) = -0.38, *p* = 0.705). However, boys were excluded slightly more often (χ^2^ (1, *N* = 10,381) = 4.93, *p* = 0.026, φ = 0.02). See Table 2 for a more detailed description of the sample by country.

**Table 1:** Sexually Explicit Material Exposure: Frequencies by Country

SEM exposure	Czechia		Finland		Malta		Poland		Portugal		Romania		Serbia		Spain		Switzerland		Total	
	***n***	**%**	***n***	**%**	***n***	**%**	***n***	**%**	***n***	**%**	***n***	**%**	***n***	**%**	***n***	**%**	***n***	**%**	***n***	**%**
Valid responses	**1805**	**83.1**	**589**	**77.9**	**772**	**80.0**	**592**	**76.4**	**1187**	**83.8**	**471**	**78.8**	**704**	**89.3**	**2071**	**92.4**	**629**	**91.3**	**8820**	**84.8**
No	792	43.9	320	54.3	442	57.3	400	67.6	752	63.4	267	56.7	244	34.7	1108	53.5	296	47.1	4621	52.4
Yes	1013	56.1	269	45.7	330	42.7	192	32.4	435	36.6	204	43.3	460	65.3	963	46.5	333	52.9	4199	47.6
Missing values	**368**	**16.9**	**167**	**22.1**	**193**	**20.0**	**183**	**23.6**	**229**	**16.2**	**127**	**21.2**	**84**	**10.7**	**170**	**7.6**	**60**	**8.7**	**1581**	**15.2**
Missing value	0	0.0	0	0.0	32	16.6	0	0.0	0	0.0	0	0.0	19	22.6	100	58.8	8	13.3	159	10.1
I don't know	187	50.8	106	63.5	80	41.5	104	56.8	111	48.5	53	41.7	34	40.5	70	41.2	24	40.0	769	48.6
Prefer not to say	181	49.2	61	36.5	81	42.0	79	43.2	118	51.5	74	58.3	31	36.9	0	0.0	28	46.7	653	41.3
Total	**2173**		**756**		**965**		**775**		**1416**		**598**		**788**		**2241**		**689**		**10,401**	

Percentages in bold represent proportion of total, and percentages in regular represent proportion of category (valid / missing)

**Table 2:** Descriptive Statistics and Reliability of Predictors and Characteristics of the Final Sample (N = 8820)

Variables	Range	Czechia	Finland	Malta	Poland	Portugal	Romania	Serbia	Spain	Switzerland
*N*		1,805	589	772	592	1,187	471	704	2,071	629
Gender [% boys]		49.2	47.9	37.3	46.8	47.3	50.0	44.9	51.7	50.1
*Descriptives [M (SD)]*										
Age	11–16	13.61 (1.72)	14.02 (1.62)	13.07 (1.51)	13.03 (1.66)	13.34 (1.59)	13.35 (1.76)	13.76 (1.73)	13.06 (1.44)	13.27 (1.41)
Time Spent Online	1–9	5.04 (2.26)	5.73 (1.86)	5.16 (2.15)	4.68 (2.19)	4.81 (2.26)	4.80 (2.46)	5.50 (2.22)	4.68 (2.30)	4.16 (1.89)
Emotional Problems	1–4	1.98 (0.73)	1.84 (0.86)	2.15 (0.84)	1.84 (0.77)	1.89 (0.73)	2.02 (0.83)	1.80 (0.74)	2.07 (0.73)	1.74 (0.68)
Sensation Seeking	1–4	1.76 (0.81)	1.75 (0.91)	1.78 (0.88)	1.59 (0.80)	1.53 (0.79)	1.68 (0.82)	1.69 (0.88)	1.62 (0.83)	1.59 (0.72)
Quality of Family Environment	1–4	3.27 (0.65)	3.44 (0.72)	3.48 (0.71)	3.41 (0.80)	3.41 (0.63)	3.30 (0.85)	3.53 (0.59)	3.47 (0.69)	3.45 (0.65)
Active Parental Mediation	1–5	2.40 (0.91)	2.53 (1.04)	3.09 (1.07)	2.58 (1.11)	2.89 (1.00)	2.90 (1.12)	3.06 (1.03)	2.73 (1.07)	2.38 (0.97)
Parental Restrictions	0–3	0.45 (0.85)	0.57 (0.89)	0.76 (1.06)	0.79 (1.09)	0.75 (1.00)	0.83 (1.13)	0.53 (0.91)	1.08 (1.12)	0.98 (1.09)
*Reliability *^*a*^										
Emotional Problems		.80	.86	.81	.81	.77	.82	.76	.71	.78
Sensation Seeking		.85	.91	.83	.85	.82	.85	.88	.87	.81
Quality of Family Environment		.68	.81	.82	.83	.74	.85	.72	.75	.77
Active Parental Mediation		.67	.83	.77	.76	.72	.76	.68	.71	.70
Restrictive Parental Mediation		.72	.66	.75	.77	.67	.80	.73	.70	.68

^**a**^ Spearman–Brown’s rho for Sensation seeking, Cronbach’s alpha for other scales

### Measures

The measures included in this paper were part of the larger EU Kids Online Project survey questionnaire, which was developed in collaboration with researchers from all participating countries (see Zlamal et al., 2020). All items included the responses ‘*I don’t know’* and ‘*Prefer not to answer*,’ which were treated as missing values. Confirmatory factor analysis (CFA) was used to test scale unidimensionality for the emotional problems measure. Other measures had three or less items, and thus, the fit of CFA models could not be evaluated. Considering the number of items in each scale, their reliability was satisfactory for each country (see Table 2 and Table 5).

*Sexually explicit materials* Questions about sexually explicit materials were framed as follows: ‘*In the PAST YEAR, you have seen lots of different images—pictures, photographs, and videos. Sometimes, these images might be obviously sexual, e.g., they may show people naked or people having sex. You might never have seen anything like this, or you may have seen something like this on a mobile phone, in a magazine, on the TV, on a DVD or on the internet. The next few questions ask you about things like this*.’ *Exposure* to such materials was assessed with one question, i.e., ‘*In the past year, have you ever seen any sexual images?’* (0 = No, 1 = Yes). Adolescents who responded ‘*Yes*’ were further asked to rate their *feelings after exposure to sexually explicit materials* (i.e., ‘*Thinking of the last time you saw images of this kind how did you feel about it?*’) on a five-point response scale (1 = *I was happy*, 2 = *I was not happy or upset*, 3 = *I was a little upset*, 4 = *I was fairly upset*, 5 = *I was very upset*). We combined responses 3–5 into one category (i.e., ‘*I was upset’*) and treated the resulting variable as categorical.

*Sensation seeking* was measured by two items, i.e., ‘*I do dangerous things for fun*,*’* and ‘*I do exciting things, even if they are dangerous’* (Slater, 2003) rated on a four-point response scale (1 = *Not true*, 2 = *A bit true*, 3 = *Fairly true*, and 4 = *Very true*). Responses were averaged across items and higher scores indicated higher levels of sensation seeking.

*Emotional problems* were measured by four items from the Emotional problems subscale of the Strengths and Difficulties Questionnaire (SDQ) (e.g., ‘*I worry a lot*,*’* ‘*I am often unhappy, sad, or tearful’*; Goodman et al., 1998) rated on a four-point response scale (1 = *Not true*, 2 = *A bit true*, 3 = *Fairly true*, and 4 = *Very true*). Responses were averaged across items and higher scores indicated higher levels of emotional problems. We examined scale structure with CFAs. Across countries, most fit indices suggested a good fit (CFI = 0.98 – 1.00, TLI = 0.94 – 1.00, SRMR = 0.01 – 0.05), though RMSEA was higher than the recommended 0.06 cutoff (Hu & Bentler, 1999) in all countries except Finland and Serbia (RMSEA = 0.04 – 0.14). However, RMSEA is inflated in models with small degrees of freedom (Kenny et al., 2015). Since this applies to our models (df = 2), we concluded that the model fits well, and the scale is unidimensional.

*Quality of family environment* was measured by three items. The first was adapted from the Health Behavior in School-aged Children survey (i.e., ‘*When I speak, someone listens to what I say*,’ WHO, 2016); the second was adapted from the Multidimensional Scale of Perceived Social Support (i.e., ‘*My family really tries to help me*’; Zimet et al., 1988); and the third was developed by the EU Kids Online network to measure perceived safety (i.e., ‘*I feel safe at home*’). All four items were rated on a four-point response scale (1 = *Not true*, 2 = *A bit true*, 3 = *Fairly true*, and 4 = *Very true*). Responses were averaged across items and higher scores indicated a higher-quality family environment.

*Active Parental mediation* of their child's internet use was measured by three items developed by the EU Kids Online network; the items assessed parents’ support of their children regarding safe internet use (e.g., ‘*Encourages me to explore and learn things on the internet*,*’* ‘*Suggests ways to use the internet safely’* (Zlamal et al., 2020). Items were rated on a five-point response scale (1 = *Never*, 2 = *Hardly ever*, 3 = *Sometimes*, 4 = *Often*, 5 = *Very often*). Responses were averaged across items and higher scores indicated more frequent use of active mediation by parents.

*Restrictive parental mediation* was assessed by asking adolescents ‘*Does your parent/carer allow you to do the following things on the internet and if so, do you need their permission to do them?’* Adolescents then responded to three items (i.e., ‘*Use a web or phone camera (*e.g., *for Skype or video chat*,*’* ‘*Download music or films*,*’* ‘*Use a social networking site (*e.g., *Facebook, Snapchat, Instagram, Twitter*’) with one of the following responses: (1) *I am allowed to do this anytime*; (2) *I am allowed to do this only with permission or supervision*; (3) *I am not allowed to do this*; (4) *I do not know if I am allowed to do this*. In this study, we dichotomized each item (response 1 was coded as 0 = *No restrictions*; responses 2 and 3 were coded as 1 = *Some restrictions*; response 4 was treated as a missing value). Responses were summed across the three dichotomized items and higher scores indicated more frequent use of restrictive mediation by parents.

*Time spent online* was assessed by asking participants to report the time they spent on the Internet during a regular weekday (i.e., a school day) on a nine-point scale (1 = *Little or no time*, 9 = *About 7 h or more*).

*Gender* was assessed by asking the participants ‘What would you say is your sex/gender?’ The options included (1) A boy, (2) A girl, (3) I don't know, and (4) Prefer not to say.

### Analysis

We conducted the analysis in two steps. First, we used binomial logistic regression to examine whether adolescents' gender, emotional problems, and sensation seeking as well as quality of family environment, active parental mediation, and parental restrictions related to the likelihood of their reported exposure to sexually explicit material. Second, we used multinomial logistic regression to examine whether gender, emotional problems, and sensation seeking as well as quality of family environment related to adolescents' odds of feeling happy or upset (both in comparison to feeling neither happy nor upset) after the exposure. In both steps, we controlled for effects of age and time spent online. All predictors (except for gender) were transformed into z-scores. Analyses were conducted in SPSS v25.0.0.1 separately for each country.

There were 43.5% of respondents with incomplete data across the examined predictors (i.e., excluding exposure to sexually explicit materials and feelings after exposure). However, only 9.0% of individual responses were missing values. Thus, to prevent reduction of statistical power and to minimize nonresponse bias, we handled missing values using multiple imputation by fully conditional specification. Following the recommendation of Leech et al. (2015), we requested 20 imputations. We included variables with no missing values (i.e., age, exposure to sexually explicit material, feelings after exposure) and country, residence size, interview mode (CASI/CAWI or PAPI), and time spent online during weekends as auxiliary variables. In conducting the imputation, logistic regressions were used to predict each item from all other items and auxiliary variables.

## Results

### Exposure to Sexually Explicit Materials

Results of the binomial regression indicated that associations between adolescents' gender, sensation seeking, and emotional problems and the likelihood of being exposed to sexually explicit materials were largely consistent across the studied countries (see Table 3 for full results). Boys were significantly more likely to report that they had encountered sexually explicit materials than girls in seven out of the nine countries (not significant in Finland and Malta). Higher sensation seeking increased the likelihood of exposure to sexually explicit materials in all nine countries. Similarly, youth who reported greater levels of emotional problems were also more likely to report that they had been exposed to sexually explicit materials in all nine countries. The results regarding our control variables (age, time spent online) were also largely consistent across countries. Older adolescents were more likely to report exposure to sexually explicit materials in all nine countries. Spending more time online increased the likelihood of exposure to sexually explicit materials in six countries (not significant in Finland, Malta, and Romania).

**Table 3:** Binomial Logistic Regressions: Odds Ratios of Variables Predicting Adolescents' Exposure to Sexually Explicit Materials (*N* = 8820)

	Czechia	Finland	Malta	Poland	Portugal	Romania	Serbia	Spain	Switzerland
	*OR*	*OR*	*OR*	*OR*	*OR*	*OR*	*OR*	*OR*	*OR*
Gender	1.68***	1.39	1.07	1.95**	2.20***	2.36***	2.22***	2.03***	1.66*
Age	1.89***	1.79***	1.52***	2.15***	1.88***	1.62***	2.10***	2.20***	2.40***
Time Spent Online	1.35***	1.22	1.21	1.29*	1.19*	1.17	1.33*	1.20**	1.41*
Emotional Problems	1.49***	1.50***	1.23**	1.41**	1.18*	1.37**	1.38**	1.38***	1.39**
Sensation Seeking	1.80***	1.60***	1.36***	1.69***	1.70***	1.47**	1.63***	1.68***	1.55***
Quality of Family Environment	0.83**	0.98	0.98	0.93	1.03	1.08	0.87	0.86*	0.92
Active Parental Mediation	1.04	0.84	0.79*	1.02	0.95	0.88	1.01	1.01	0.99
Parental Restrictions	0.76**	0.90	0.88	0.95	0.77**	0.73**	0.81	0.76***	0.95
Constant	0.80*	0.48***	0.73**	0.38***	0.39***	0.48***	1.32	0.73***	1.22
*Pseudo-R*^*2*^									
Cox & Snell	.27	.23	.14	.24	.21	.20	.27	.27	.23
Nagelkerke	.36	.30	.18	.33	.29	.27	.37	.35	.31
*Model Chi-square Test *^*a*^									
χ^2^	545.67–575.10	144.69–157.36	100.77–120.49	152.67–167.95	278.35–290.87	98.81–113.33	217.07–225.71	624.21–656.14	163.53–173.47
df	8	8	8	8	8	8	8	8	8
*p*	< .001	< .001	< .001	< .001	< .001	< .001	< .001	< .001	< .001

^a^ we present the range of χ^2^ values across the 20 imputations, *p < .05, ** *p* < .01, *** *p* < .001

On the other hand, the results regarding parental restrictions, quality of family environment, and active parental mediation were mixed, and we detected their effects only in a minority of the studied countries. Higher levels of parental restrictions were linked to a lower likelihood of exposure to sexually explicit materials in four out of the nine countries (Czechia, Portugal, Romania, and Spain); no significant relationship was found in the other five countries. A higher quality of family environment decreased the likelihood of exposure to sexually explicit materials in Czechia and Spain. More active parental mediation decreased the likelihood of exposure to sexually explicit materials in Portugal. These relationships were not significant in the other studied countries.

### Feelings after Exposure to Sexually Explicit Materials

The second step of our analysis was conducted on 3,364 adolescents aged 11–16 (M_age_ = 14.04, SD = 1.50, 54.3% male), who reported that they were exposed to sexually explicit materials and provided responses about how they felt after exposure (see Table 4). As apparent from the first step of our analysis, this is a specific population that differed from a general adolescent population (see Table 5 for sample characteristics). In the multinomial logistic regression, we used the response ‘I felt neither happy nor upset’ (we use the term *neutral* for brevity) as the reference category.

**Table 4:** Feelings After Exposure to Sexually Explicit Materials: Frequencies by Country

Feelings After SEM Exposure	Czechia		Finland		Malta		Poland		Portugal		Romania		Serbia		Spain		Switzerland		Total	
	***n***	**%**	***n***	**%**	***n***	**%**	***n***	**%**	***n***	**%**	***n***	**%**	***n***	**%**	***n***	**%**	***n***	**%**	***n***	**%**
Valid Responses	**790**	**78.0**	**222**	**82.5**	**225**	**68.2**	**132**	**68.8**	**334**	**76.8**	**177**	**86.8**	**386**	**83.9**	**839**	**87.1**	**259**	**77.8**	**3364**	**80.1**
I was happy	228	28.9	63	28.4	59	26.2	26	19.7	105	31.4	29	16.4	103	26.7	331	39.5	36	13.9	980	29.1
I was not happy or upset	302	38.2	110	49.5	72	32.0	59	44.7	162	48.5	66	37.3	183	47.4	311	37.1	75	29.0	1340	39.8
I was upset	260	32.9	49	22.1	94	41.8	47	35.6	67	20.1	82	46.3	100	25.9	197	23.5	148	57.1	1044	31.0
Missing Values	**223**	**22.0**	**47**	**17.5**	**105**	**31.8**	**60**	**31.3**	**101**	**23.2**	**27**	**13.2**	**74**	**16.1**	**124**	**12.9**	**74**	**22.2**	**835**	**19.9**
Missing value	5	2.2	1	2.1	15	14.3	0	0.0	0	0.0	0	0.0	7	9.5	41	33.1	14	18.9	83	9.9
I don't know	140	62.8	40	85.1	69	65.7	44	73.3	59	58.4	16	59.3	51	68.9	83	66.9	42	56.8	544	65.1
Prefer not to say	78	35.0	6	12.8	21	20.0	16	26.7	42	41.6	11	40.7	16	21.6	0	0.0	18	24.3	208	24.9
Total	**1013**		**269**		**330**		**192**		**435**		**204**		**460**		**963**		**333**		**4199**	

Percentages in bold represent proportion of total, and percentages in regular represent proportion of category (valid / missing)

**Table 5:** Descriptive Statistics and Reliability of Predictors and Characteristics of the Subsample of Adolescents who Reported Feelings After Exposure to Sexually Explicit Materials (N = 3364)

	Range	Czechia	Finland	Malta	Poland	Portugal	Romania	Serbia	Spain	Switzerland
*N*		790	222	225	132	334	177	386	839	259
Gender [% boys]		53.42	47.57	41.33	55.30	58.98	60.23	51.04	59.12	52.51
*Descriptives [M (SD)]*										
Age	11–16	14.18 (1.60)	14.67 (1.30)	13.6 (1.61)	14.09 (1.67)	14.16 (1.40)	13.95 (1.67)	14.34 (1.49)	13.74 (1.36)	13.88 (1.19)
Time Spent Online	1–9	5.64 (2.16)	6.16 (1.67)	5.54 (1.96)	5.56 (2.21)	5.36 (2.09)	5.25 (2.29)	6.02 (2.02)	5.37 (2.19)	4.73 (1.71)
Emotional Problems	1–4	2.12 (0.74)	2.11 (0.91)	2.30 (0.88)	2.08 (0.87)	1.98 (0.74)	2.15 (0.82)	1.86 (0.77)	2.25 (0.75)	1.88 (0.70)
Sensation Seeking	1–4	2.00 (0.85)	2.09 (0.95)	2.04 (0.92)	1.98 (0.93)	1.88 (0.89)	1.96 (0.87)	1.87 (0.92)	1.92 (0.92)	1.76 (0.72)
Quality of Family Environment	1–4	3.22 (0.66)	3.38 (0.76)	3.36 (0.74)	3.24 (0.85)	3.39 (0.60)	3.31 (0.83)	3.51 (0.59)	3.37 (0.72)	3.39 (0.67)
*Reliability *^*a*^										
Emotional Problems		.78	.86	.83	.83	.78	.82	.79	.71	.78
Sensation Seeking		.85	.90	.86	.88	.80	.85	.88	.88	.78
Quality of Family Environment		.70	.85	.80	.82	.72	.85	.70	.76	.79

^**a**^ Spearman–Brown’s rho for Sensation seeking, Cronbach’s alpha for other scales

Results indicated that across different country contexts, gender consistently played an important role in feelings after exposure (see Table 7 for frequencies) to sexually explicit content (see Table 6 for full results). Boys were more likely to feel happy than neutral in all studied countries and they were also less likely to feel upset than neutral in seven out of the nine studied countries (not significant in Poland and Spain). However, the analysis yielded suspiciously large odds ratios for the effects of gender in most countries. This was likely caused by the low number of girls (*n* = 128, 8.3%, see Table 7 for country results) who reported feeling happy after exposure to sexually explicit materials. Thus, the odds ratios were probably inflated, overemphasizing the role of gender. Odds ratios in countries, where at least 10 girls reported feeling happy after exposure to sexually explicit materials (i.e., Czechia, Malta, and Spain) likely provide more realistic estimates. Analogously, only four Finnish boys (4.0%) reported feeling upset leading to an unrealistically small odds ratio for that country context.

**Table 6:** Multinomial Logistic Regression: Odds Ratios of Variables Predicting Adolescents' Feelings After Exposure to Sexually Explicit Materials (N = 3364)

	Czechia	Finland	Malta	Poland	Portugal	Romania	Serbia	Spain	Switzerland
	*OR*	*OR*	*OR*	*OR*	*OR*	*OR*	*OR*	*OR*	*OR*
*Happy (vs. Neutral) *^*a*^									
Intercept	***	***	**	**	***	**	***	***	**
Age	1.23	1.17	1.22	0.64	0.76	0.86	1.57**	0.89	2.09
Time Spent Online	1.10	0.61	1.15	1.23	1.45*	1.66	1.23	1.13	0.91
Emotional Problems	0.91	1.01	0.84	1.12	0.85	1.58	0.97	0.88	1.34
Sensation Seeking	1.37**	1.38	1.66*	1.77*	1.54**	1.20	1.43*	1.18*	1.70*
Quality of Family Environment	0.96	0.53**	0.94	0.70	0.78	0.70	1.31	0.88	0.63*
Gender = male	13.20***	10.08***	3.37**	26.41**	11.86***	32.05**	44.65***	3.97***	27.06**
*Upset (vs. Neutral) *^*a*^									
Intercept					**	**		***	***
Age	0.54***	0.48**	0.47***	0.64*	0.43***	0.66*	0.50***	0.50***	0.53**
Time Spent Online	0.74**	0.61	1.24	0.97	0.88	0.85	0.80	1.01	0.74
Emotional Problems	1.31*	1.19	1.13	1.27	1.35	1.28	1.42*	1.19	1.28
Sensation Seeking	0.75**	0.68	0.93	1.02	0.80	0.92	0.69*	0.81*	0.71
Quality of Family Environment	1.10	0.84	1.03	0.98	0.78	1.06	1.37	0.97	1.39
Gender = male	0.67*	0.08***	0.40*	0.69	0.37**	0.28**	0.23***	0.82	0.19***
*Pseudo-R*^*2*^									
Cox & Snell	.36	.43	.32	.29	.35	.29	.45	.19	.38
Nagelkerke	.40	.49	.36	.33	.40	.33	.51	.21	.44
*Model fit *^*b*^									
χ^2^	340.87–355.43	117.18–129.44	85.08–91.61	42.81–47.27	139.36–144.74	58.39–64.11	228.60–233.36	168.07–178.14	119.51–125.97
df	12	12	12	12	12	12	12	12	12
*p*	< .001	< .001	< .001	< .001	< .001	< .001	< .001	< .001	< .001

^a^ response *I felt neither happy nor upset* was used as reference category, ^b^ we present the range of χ^2^ values across the 20 imputations, ^*^ *p* < .05, ** *p* < .01, *** *p* < .001

**Table 7:** Adolescents' Feelings After Exposure to Sexually Explicit Materials: Frequencies by Gender and Country

Feelings after SEM exposure	Czechia		Finland		Malta		Poland		Portugal		Romania		Serbia		Spain		Switzerland	
	*N*	%	*N*	%	*N*	%	*N*	%	*N*	%	*N*	%	*N*	%	*N*	%	*N*	%
*Boys*																		
I was happy	207	49.1	55	52.0	43	46.2	25	34.2	97	49.2	28	26.3	99	50.3	263	53.0	35	25.7
I was not happy or upset	127	30.1	47	44.1	30	32.3	29	39.7	80	40.6	46	43.2	78	39.6	150	30.2	53	39.0
I was upset	88	20.9	4	4.0	20	21.5	19	26.0	20	10.2	33	30.6	20	10.2	83	16.7	48	35.3
**Total**	**422**		**106**		**93**		**73**		**197**		**107**		**197**		**496**		**136**	
*Girls*																		
I was happy	21	5.7	8	7.0	16	12.1	1	1.7	8	5.8	1	1.4	4	2.1	68	19.8	1	0.8
I was not happy or upset	175	47.6	64	54.6	42	31.8	30	50.8	82	59.9	20	28.4	105	55.6	161	46.9	22	17.9
I was upset	172	46.7	45	38.6	74	56.1	28	47.5	47	34.3	49	70.2	80	42.3	114	33.2	100	81.3
Total	**368**		**116**		**132**		**59**		**137**		**70**		**189**		**343**		**123**	

The effect of sensation seeking was also consistent across countries, but not for both happy and upset feelings. Although adolescents with higher sensation seeking were more likely to feel happy rather than neutral in seven countries (not significant in Finland and Romania), there was lower likelihood of them feeling upset after exposure to sexually explicit materials only in three countries—Czechia, Serbia, and Spain (not significant in other countries). Other relationships were detected only in certain countries. Emotional problems had no effect on the likelihood of feeling happy after exposure to sexually explicit materials in any country but increased the likelihood of feeling sad in both Czechia and Serbia. Better quality of family environment decreased the likelihood of feeling happy after exposure in Finland and Switzerland but had no effect on the likelihood of feeling upset in any other country.

Regarding our control variables, older age increased the likelihood of feeling happy after exposure to sexually explicit materials only in Serbia (not significant in other countries). However, older adolescents were less likely to report feeling upset after exposure in all nine countries. More time spent online increased adolescents' likelihood of feeling happy after exposure to sexually explicit materials in Serbia and decreased their likelihood of feeling upset in Czechia; there were no significant relations in the other countries.

## Discussion

The present study explored the associations between adolescents’ individual and social characteristics and the likelihood that they had been exposed to sexually explicit materials (see RQ1). It is also the first to use a quantitative approach to investigate the association of these characteristics with positive and negative feelings after exposure to sexually explicit content (see RQ2). Additionally, much of the extant work on adolescents’ exposure to sexually explicit material has been carried out with country-specific samples. To determine whether the underlying relationships between various individual and family variables vary by context, the present study used a cross-national approach involving nine European countries.

### Adolescents’ Exposure to Sexually Explicit Materials

Exposure to sexually explicit materials in our study varied from 32 to 65%, and on average, 48% of adolescents reported that they had seen some sexually explicit materials in the past year, online or offline. This prevalence is slightly lower than that reported in previous research (see, for example, Andrie et al., 2021 who asked adolescents about sexually explicit media and Donevan et al., 2021 who inquired about watching pornography). One possible explanation for the lower rates in our sample might be that the mean age of our sample was 14.04 years old, whereas both of the other studies sampled high schoolers, and exposure to sexually explicit materials in known to increase with age (Ševčíková et al., 2014; Perry & Heyward, 2017; Ma et al., 2017a).

### Individual Characteristics Related to Exposure to Sexually Explicit Materials

Boys in our sample had a greater likelihood of exposure, a finding that is consistent with that of prior research (Beyens et al., 2015; Ševčíková et al., 2014; Vandenbosch, 2015; Vandenbosch & Peter, 2016). Researchers have speculated that this gender difference is likely because boys report earlier sexual activity (e.g., masturbation) and due to prevalent gender stereotypes (Ševčíková & Daneback, 2014). Although this was found broadly in most of the country contexts of our sample, we did not find an association between gender and exposure to sexually explicit materials in two countries. In a later section, we discuss the cross-national implications of the study results.

Consistent with prior research (Beyens et al., 2015; Peter & Valkenburg, 2006; Ševčíková et al., 2014; Vandenbosch, 2015), sensation seekers had a significantly higher likelihood of being exposed to sexually explicit materials in all countries. The most likely explanation for this finding is that for youth who are high in sensation seeking, sexually explicit content may be novel, exciting, and shocking; additionally, as something that may be viewed as forbidden, sensation seekers might find the idea of accessing pornography as rule-breaking and thus exciting.

Adolescents with emotional problems also had a significantly higher likelihood of exposure to sexually explicit materials in all nine countries, a finding that is also consistent with the majority of prior work (Doornwaard et al., 2016; Tsitsika et al,. 2009; Ybarra & Mitchell, 2005). Kohut and Štulhofer (2018) have noted that just as adults have been found to use pornography to improve mood and adjust to psychological difficulties, such as depression (Bridges & Morokoff, 2010), adolescents with emotional problems may also access sexually explicit materials to deal with their emotional problems. An alternative possibility is that consumption of pornography may lead to emotional problems; however, one longitudinal study found no evidence for this among boys and limited contradictory evidence among girls (Kohut & Štulhofer, 2018).

### Social Characteristics Important for Exposure to Sexually Explicit Materials

Our results suggested no relationship between the quality of family environment and likelihood of exposure in most of the countries. This contrasts with earlier research, which showed that family variables may prevent exposure (Ma et al., 2017a, 2017b; Mesch, 2009; Shek & Ma, 2012; Ybarra & Mitchell, 2005). Two of the prior studies were conducted in China, and there are no recent studies which have focused on the quality of family environment within Western contexts. Thus, it is possible that the quality of the family environment does not serve a protective role within the European context, where exposure to sexually explicit materials has become much more normalized among adolescents (Scott et al., 2020; Steinberg et al., 2019). Additionally, the mean age of our sample was 14.04 years versus a mean age of 12.43 and 12.6 years for the Chinese sample (Ma et al., 2017b; Shek & Ma, 2012); so, it may be that the quality of the family environment is negatively associated with exposure to sexually explicit materials during early adolescence when accessing sexually explicit content is less developmentally normative.

This is one of the first studies to investigate active and restrictive parental mediation of technology use in conjunction with other individual and family characteristics in nine countries, and we found mixed results. We did not find a role for active parental mediation in most countries. Restrictive parental mediation of technology use was negatively related to exposure to sexually explicit materials in four countries, but there was no relationship in the other five. The lack of relationship between active parental mediation and exposure to sexually explicit materials in most countries may be because even though parents actively mediate technology use, they do not focus on topics related to sexuality. Recent qualitative studies suggest that whereas parents believe that adolescents and children are exposed to sexually explicit materials in general, they think their own child is not (Davis et al., 2019; Zurcher, 2017). On the other hand, restrictive parental mediation of technology use prevents a range of positive and negative activities (Livingstone et al., 2017a, 2017b), which might explain why it was associated with lower exposure in some countries. Future studies should examine the use and effectiveness of general and specific parental mediation approaches in preventing exposure to sexually explicit materials.

### Feelings after Exposure to Sexually Explicit Materials

The present study is one of the first to quantify adolescents’ feelings after exposure to sexually explicit materials and to examine the characteristics associated with feeling happy and upset after exposure. Across all countries, being a boy was associated with feeling happy after viewing sexually explicit materials. In most of the countries, being a boy also meant a greater likelihood of feeling neutral and not upset after the exposure. These results are similar to those of qualitative studies, which have found gender differences in feelings after exposure and in particular that girls experience more negative feelings after exposure to sexually explicit materials (Cameron et al., 2005; Doornwaard et al., 2016; Lewis et al., 2018). There are several possible explanations for why boys and girls report different feelings after exposure to sexually explicit content. First, boys consume sexually explicit materials more frequently than girls, and for different reasons; boys reported that their primary reason to watch pornography was arousal, whereas for girls, it was curiosity (Ševčíková & Daneback, 2014). Thus, boys may watch pornography more often with the expectation of becoming aroused, leaving them happy afterward. Second, girls may be more disturbed when they are unintentionally exposed to sexually explicit materials. Prior studies have not found a link between gender and unwanted exposure to sexually explicit materials (Mitchell et al., 2014; Ševčíková et al., 2014), but it is perhaps more distressing for girls to experience solicitation and receive unwanted nude photographs—whether of strangers, peers, or of their female friends. For example, in a qualitative study by Lewis et al. (2018), girls reported that they experienced sexual images of other girls being circulated on social networks by their peers and felt it was ‘unacceptable.’ Such findings are also echoed in the research of Ringrose et al. (2012), who investigated peer culture around sexting. Third, mainstream pornography often sexually objectifies women (Fritz & Paul, 2017), which may be especially distressing for young girls to see, as they are more likely to identify with the sexually objectified women on screen compared to boys (Cohen, 2001; Wright, 2011). More research is needed to tease apart the relative roles of maturational/sexual development and contextual pressures (e.g., sexualization of adolescent girls in media) with regard to the gender differences in feelings after exposure to sexually explicit content.

### Individual Characteristics Related to Feelings after Exposure to Sexually Explicit Materials

Prior work suggested that high sensation seekers may perceive encountering risky content as more pleasurable, and thus, seeing more risky content may make them more resilient. Conversely, those who are not sensation seekers are likely to be left more upset after seeing such content (Livingstone & Görzig, 2014). Extending this idea to the underlying mechanism, we expected sensation seeking to act as a buffer from negative feelings and an amplifier of positive feelings. In accordance with our expectations, high sensation seeking was positively associated with the likelihood of feeling happy after seeing sexually explicit materials in most of the countries. However, high sensation seeking was related to a lower likelihood of feeling upset after exposure to sexually explicit materials in only three countries. The results suggest that although high sensation seeking was generally associated with positive emotions, the buffering role of sensation seeking from sexually explicit content may not be universal. Future research should explore whether the role of sensation seeking may be moderated by whether exposure to sexually explicit materials is intentional or not.

In contrast to the theoretical idea that adolescents with emotional difficulties may be more vulnerable to experiencing harm (Livingstone & Görzig, 2014), we did not find a relationship between emotional problems and the likelihood of feeling upset in a majority of the countries. We also did not find any association for emotional problems and feeling happy after seeing sexually explicit materials. This could be because other characteristics were at play, such as different content of the materials or the (un)intentionality of the exposure. We speculate that exposure to preferred content while having emotional problems does not result in negative feelings but seeing disturbing content while having emotional problems may worsen the feelings. Future research is needed to explore these alternative possibilities.

### Social Characteristics Associated with Feelings after Exposure to Sexually Explicit Materials

Prior research suggests that the quality of the family environment may also protect adolescents from the possible harm that may come from exposure (Löfgren-Mårtenson & Månsson, 2010). Surprisingly, we did not find an association between family quality and feeling upset after viewing sexually explicit materials. In most countries, quality of the family environment was not associated with feeling happy except for Finland and Switzerland; in these two countries, good quality family environment was negatively associated with the odds of feeling happy after exposure. More research is needed to uncover the social characteristics underlying adolescents’ feeling after exposure.

### Age and Feelings after Exposure to Sexually Explicit Materials

In all countries, age was negatively related to the likelihood of feeling upset. With regard to positive feelings there was no association with age, except for Serbia. These results are consistent with prior research, which suggests that negative feelings after exposure to sexually explicit materials decrease with increasing age (Martellozzo et al., 2020). This might be explained by the desensitization and normalization of exposure to sexually explicit materials during adolescence: as youth grow older, they become more familiar with sexually explicit content, and it is not as shocking for them because they know what to expect from it (Daneback et al., 2018). At the same time, it does not mean that they feel more positive about such content as they get older.

### Implications of Cross-national Findings: Country-Level Trends

An important feature of our study was that the results are based on data from nine different European countries. Overall, they showed that although there were some differences between the countries in terms of rates of exposure to sexually explicit material and feelings on exposure, there were many similarities in the underlying relationships between the study variables, such as age, gender, individual characteristics and family support and parental mediation.

Our findings that youth in the different country contexts were actually more similar to each other than different with regard to key study variables is consistent with findings from prior cross-country European research (Ševčíková et al., 2014) and offline developmental trends in sexuality (Cubbin et al., 2005; Juhasz et al., 1986), as well as the research on offline problem behavior (alcohol use, early sexual behavior) (Vazsonyi et al., 2008) and online harmful content (Kvardova et al., 2021). These studies have found more cross-national similarities than differences in the explanatory role of risk and protective factors. Similar findings have been reported in cross-cultural psychological research (Fischer & Schwartz, 2011) and Schwartz (2014, p. 2) notes that ‘values vary much more within countries than between countries.’ Greenfield (2014) has proposed that the similarity in values between countries may be because sociodemographic factors within countries drive differences between values—specifically, globalization, urbanization, proliferation of the internet, and formal education systems are widespread in the Western world. Thus, urban, educated, and higher-income individuals in different western countries may be more similar to each other in values than they are to individuals within their own countries who may not be as educated or have access to the same level of income and technologies.

Another area of similarity among adolescents in Western individualized countries may be that of developmental tasks, which are the tasks that individuals are supposed to master during different life periods including adolescence (e.g., identity and sexuality) and adulthood (e.g., careers) (Havighurst, 1948). Developmental tasks vary by culture and time, and the tasks facing twenty-first-century adolescents in Western countries are different from those of earlier eras. An important developmental task relevant to youth today is that of sexual development, which entails adjusting to their maturing bodies and growing sexual feelings (Jugert & Titzman, 2020; Simpson, 2001). Extant research suggests considerable similarity among adolescents in the western world with regard to the importance of their developing sexuality and their use of online spaces in the service of this important developmental task (Alexandraki et al., 2018; Harden, 2014; Owens et al., 2012; Scott et al., 2020). Thus, it may be that youth from different country contexts are similar in their exposure to sexually explicit content because similar sociodemographic factors are driving their interest, access, and reactions to such content.

Although findings suggest that adolescents in the study sample were similar across European countries, we also found some differences. An in-depth examination of these differences is beyond the scope of this paper, and we briefly examine the factors that may be at play. We speculate that the differences may be explained by broader societal, economic, and media use-related factors, which create specific environments, which may alter the outcomes of media use. Among these factors, for example, are quality and place of internet access and policies oriented toward the well-being of children and adolescents, both closely tied to digital skills and internet safety (Staksrud & Milosevic, 2017). Livingstone et al., (2017a, 2017b) present a model of children’s internet use, which suggests that societal (i.e., family, educators, peers, digital ecology) and country variables (i.e., culture, media and values, societal inclusion, or education and knowledge) affect outcomes of technology use. We suggest that future research make use of these models and include the newest findings from both developed Western countries, such as the USA, but also from non-Western perspectives to better understand the factors that drive these differences (Staksrud & Milosevic, 2017). Our study design limits our ability to address the obtained cross-national differences and this issue is discussed further within the limitations section.

### Limitations

There are several limitations to the present study that should be taken into consideration. Our measures of active and restrictive parental mediation were focused on technology use in general, and future studies in this area should assess parental mediation specifically related to online sexually explicit materials. Our measure of exposure to sexually explicit material was dichotomous (see Kohut et al., 2020 and Fisher & Kohut, 2020, for further information); future studies would benefit from employing a frequency scale. We also did not distinguish between intentional and unintentional exposure to sexually explicit materials, an issue that might play a role in how adolescents feel when they are exposed to it. Additionally, the type of content youth had seen and whether they liked it might also affect their feelings. There may also be mediating variables, such as moral disapproval or religiosity. The research by Martellozzo and colleagues (2020) also suggests that adolescents experience a whole range of emotions after exposure, and thus, measuring feelings on the range from happy to upset may be too narrow. We also did not distinguish between a participants’ first exposure to sexually explicit materials ever, or a repeated one. Feelings after the first exposure presumably differs from feelings after the last or repeated exposure (Martellozzo et al., 2020). Future research should investigate these differences and explore the moderating role of familiarity with sexual content. For the questions about exposure to sexually explicit materials and their subsequent feelings, participants were asked to consider their experiences over a one year period. This is an important limitation, as we can only analyze feelings after exposure per se and draw no conclusions about the impact of exposure to sexually explicit materials on adolescents. Future research might benefit from a shorter period of self-reported time or from a different method of collection, such as ecological momentary assessment that allows researchers to assess experiences over a much shorter time period and potentially yielding a more accurate measurement. Finally, we conducted the regression models for each country separately, and thus, we cannot directly compare the results between countries (for example, in terms of effect sizes). Although our study found many similarities, there were some differences, and our study design does not allow us to provide an explanation for them, limiting the applicability of our findings. Future research which compares countries should consider using different statistical approaches, such as the multilevel analysis, that could help unravel the reason for cross-country differences.

### Implications and Conclusions

The results of this study have important theoretical and practical implications. Our study’s results add to the theoretically significant finding that similar relationships between variables such as sensation seeking and emotional problems may predict a range of adolescent outcomes including offline problem behavior (Vazonyi, 2008), accessing harmful online content (Kvardova et al., 2021), and accessing online sexually explicit material. Additionally, our results revealed that active parental mediation is not associated with exposure to sexually explicit materials in most countries, and that the evidence for a relationship between restrictive parental mediation and exposure is mixed. We found striking gender differences in feelings after exposure: compared to girls, boys more often reported feeling happy, whereas girls more often reported feeling upset. However, the majority of adolescents felt neutral after exposure, which suggests that seeing sexually explicit materials is not as distressing as originally thought. Our findings are important for practitioners, educators, and policymakers, as they can help to identify which adolescents may be more likely to be exposed to sexual materials and/or be upset after exposure. Regarding caregivers, restricting adolescents’ access to technology in general is not always effective in preventing exposure to sexualized content, and active mediation of technology use is ineffective too. More studies are needed to understand factors that may drive national differences in the effectiveness of restrictive mediation. With regard to designing interventions, studies should uncover whether specific mediation of sexual content is more effective than general mediation of technology use. The present study is a first step, and it is expected that future research will further examine adolescents’ feelings after exposure to sexually explicit materials, the underlying factors related to them (e.g., sensation seeking), and the long-term implications of exposure for adolescent sexual development and well-being.

## Supplementary Information

Below is the link to the electronic supplementary material.

Supplementary file1 (DOCX 41 KB)
