# Supplementary material for: Exposure to Sexually Explicit Materials and Feelings after Exposure among Adolescents in Nine European Countries: The Role of Individual Factors and Social Characteristics
Source: Arch Sex Behav. 2022 Aug 29;52(1):333–51. doi: 10.1007/s10508-022-02401-9 (PMC9859855; doi:10.1007/s10508-022-02401-9)
Supplement: Supplementary file 1 — Supplementary file1 (DOCX 41 KB) [file 10508_2022_2401_MOESM1_ESM.docx]

# Supplemental tables

**Supplemental Table 1**: Full Results of Binomial Logistic Regression (SEM Exposure): Czechia, Finland, Malta.

| **Predictors** | **Czechia (*N* = 1805)** | | | | **Finland (*N* = 589)** | | | | **Malta (*N* = 772)** | | | |
| --- | --- | --- | --- | --- | --- | --- | --- | --- | --- | --- | --- | --- |
|  | ***B* (*SE*)** | ***p*** | ***OR*** | **95% CI** | ***B* (*SE*)** | ***p*** | ***OR*** | **95% CI** | ***B* (*SE*)** | ***p*** | ***OR*** | **95% CI** |
| Gender | 0.52 (0.12) | <.001 | 1.68 | [1.33, 2.14] | 0.33 (0.22) | 0.129 | 1.39 | [0.91, 2.12] | 0.07 (0.17) | 0.685 | 1.07 | [0.77, 1.50] |
| Age | 0.64 (0.06) | <.001 | 1.89 | [1.66, 2.14] | 0.58 (0.11) | <.001 | 1.79 | [1.43, 2.22] | 0.42 (0.09) | <.001 | 1.52 | [1.26, 1.82] |
| Time Spent Online | 0.30 (0.06) | <.001 | 1.35 | [1.19, 1.54] | 0.20 (0.13) | 0.125 | 1.22 | [0.95, 1.57] | 0.19 (0.10) | 0.057 | 1.21 | [0.99, 1.46] |
| Emotional Problems | 0.40 (0.07) | <.001 | 1.49 | [1.30, 1.70] | 0.41 (0.10) | <.001 | 1.50 | [1.23, 1.84] | 0.21 (0.08) | 0.009 | 1.23 | [1.05, 1.44] |
| Sensation Seeking | 0.59 (0.07) | <.001 | 1.80 | [1.57, 2.07] | 0.47 (0.10) | <.001 | 1.60 | [1.32, 1.94] | 0.31 (0.09) | <.001 | 1.36 | [1.15, 1.62] |
| Quality of Family Environment | -0.19 (0.07) | 0.006 | 0.83 | [0.73, 0.95] | -0.02 (0.10) | 0.838 | 0.98 | [0.80, 1.20] | -0.02 (0.09) | 0.826 | 0.98 | [0.82, 1.17] |
| Active Parental Mediation | 0.04 (0.07) | 0.583 | 1.04 | [0.90, 1.20] | -0.18 (0.12) | 0.124 | 0.84 | [0.66, 1.05] | -0.24 (0.09) | 0.010 | 0.79 | [0.65, 0.94] |
| Parental Restrictions | -0.27 (0.09) | 0.002 | 0.76 | [0.64, 0.91] | -0.11 (0.14) | 0.430 | 0.90 | [0.68, 1.18] | -0.13 (0.10) | 0.185 | 0.88 | [0.73, 1.06] |
| Constant | -0.22 (0.09) | 0.013 | 0.80 | [0.68, 0.96] | -0.73 (0.16) | <.001 | 0.48 | [0.35, 0.67] | -0.31 (0.12) | 0.006 | 0.73 | [0.58, 0.92] |

**Supplemental Table 2**: Full Results of Binomial Logistic Regression (SEM Exposure): Poland, Portugal, Romania.

| **Predictors** | **Poland (*N* = 592)** | | | | **Portugal (*N* = 1187)** | | | | **Romania (*N* = 471)** | | | |
| --- | --- | --- | --- | --- | --- | --- | --- | --- | --- | --- | --- | --- |
|  | ***B* (*SE*)** | ***p*** | ***OR*** | **95% CI** | ***B* (*SE*)** | ***p*** | ***OR*** | **95% CI** | ***B* (*SE*)** | ***p*** | ***OR*** | **95% CI** |
| Gender | 0.67 (0.22) | .002 | 1.95 | [1.27, 2.98] | 0.79 (0.15) | <.001 | 2.20 | [1.64, 2.94] | 0.86 (0.23) | <.001 | 2.36 | [1.49, 3.73] |
| Age | 0.77 (0.11) | <.001 | 2.15 | [1.74, 2.67] | 0.63 (0.08) | <.001 | 1.88 | [1.61, 2.19] | 0.48 (0.11) | <.001 | 1.62 | [1.31, 1.98] |
| Time Spent Online | 0.25 (0.12) | .033 | 1.29 | [1.02, 1.62] | 0.17 (0.08) | .025 | 1.19 | [1.02, 1.38] | 0.15 (0.11) | .147 | 1.17 | [0.95, 1.44] |
| Emotional Problems | 0.35 (0.11) | .001 | 1.41 | [1.15, 1.74] | 0.17 (0.08) | .034 | 1.18 | [1.01, 1.38] | 0.32 (0.11) | .005 | 1.37 | [1.10, 1.71] |
| Sensation Seeking | 0.52 (0.11) | <.001 | 1.69 | [1.36, 2.09] | 0.53 (0.08) | <.001 | 1.70 | [1.45, 1.98] | 0.38 (0.12) | .002 | 1.47 | [1.15, 1.87] |
| Quality of Family Environment | -0.07 (0.10) | .444 | 0.93 | [0.77, 1.12] | 0.03 (0.09) | .758 | 1.03 | [0.87, 1.21] | 0.07 (0.10) | .457 | 1.08 | [0.89, 1.31] |
| Active Parental Mediation | 0.02 (0.12) | .849 | 1.02 | [0.81, 1.29] | -0.05 (0.08) | .523 | 0.95 | [0.81, 1.11] | -0.13 (0.11) | .265 | 0.88 | [0.71, 1.10] |
| Parental Restrictions | -0.05 (0.12) | .643 | 0.95 | [0.75, 1.19] | -0.26 (0.09) | .004 | 0.77 | [0.65, 0.92] | -0.32 (0.12) | .007 | 0.73 | [0.58, 0.92] |
| Constant | -0.98 (0.15) | <.001 | 0.38 | [0.28, 0.51] | -0.94 (0.11) | <.001 | 0.39 | [0.32, 0.48] | -0.74 (0.17) | <.001 | 0.48 | [0.34, 0.67] |

**Supplemental Table 3**: Full Results of Binomial Logistic Regression (SEM Exposure): Serbia, Spain, Switzerland.

| **Predictors** | **Serbia (*N* = 704)** | | | | **Spain (*N* = 2071)** | | | | **Switzerland (*N* = 629)** | | | |
| --- | --- | --- | --- | --- | --- | --- | --- | --- | --- | --- | --- | --- |
|  | ***B* (*SE*)** | ***p*** | ***OR*** | **95% CI** | ***B* (*SE*)** | ***p*** | ***OR*** | **95% CI** | ***B* (*SE*)** | ***p*** | ***OR*** | **95% CI** |
| Gender | 0.80 (0.22) | <.001 | 2.22 | [1.46, 3.38] | 0.71 (0.11) | <.001 | 2.03 | [1.63, 2.53] | 0.51 (0.20) | .012 | 1.66 | [1.12, 2.46] |
| Age | 0.74 (0.11) | <.001 | 2.10 | [1.70, 2.60] | 0.79 (0.07) | <.001 | 2.20 | [1.93, 2.51] | 0.87 (0.13) | <.001 | 2.40 | [1.85, 3.11] |
| Time Spent Online | 0.28 (0.11) | .013 | 1.33 | [1.06, 1.65] | 0.18 (0.06) | .002 | 1.20 | [1.07, 1.34] | 0.35 (0.14) | .010 | 1.41 | [1.09, 1.84] |
| Emotional Problems | 0.32 (0.11) | .005 | 1.38 | [1.10, 1.72] | 0.32 (0.06) | <.001 | 1.38 | [1.22, 1.55] | 0.33 (0.12) | .004 | 1.39 | [1.11, 1.75] |
| Sensation Seeking | 0.49 (0.12) | <.001 | 1.63 | [1.29, 2.04] | 0.52 (0.06) | <.001 | 1.68 | [1.49, 1.89] | 0.44 (0.12) | <.001 | 1.55 | [1.21, 1.97] |
| Quality of Family Environment | -0.14 (0.12) | .274 | 0.87 | [0.68, 1.11] | -0.15 (0.06) | .015 | 0.86 | [0.76, 0.97] | -0.08 (0.11) | .455 | 0.92 | [0.75, 1.14] |
| Active Parental Mediation | 0.01 (0.11) | .933 | 1.01 | [0.81, 1.25] | 0.01 (0.06) | .939 | 1.01 | [0.89, 1.13] | -0.01 (0.11) | .951 | 0.99 | [0.80, 1.24] |
| Parental Restrictions | -0.21 (0.13) | .106 | 0.81 | [0.63, 1.05] | -0.27 (0.06) | <.001 | 0.76 | [0.67, 0.86] | -0.05 (0.11) | .642 | 0.95 | [0.76, 1.19] |
| Constant | 0.28 (0.14) | .051 | 1.32 | [1.00, 1.76] | -0.31 (0.08) | <.001 | 0.73 | [0.62, 0.87] | 0.20 (0.15) | .171 | 1.22 | [0.92, 1.62] |

**Supplemental Table 4**: Full Results of Multinomial Logistic Regression (Feelings After SEM Exposure): Czechia, Finland, Malta.

| **Predictors** | **Czechia (*N* = 790)** | | | | **Finland (*N* = 222)** | | | | **Malta (*N* = 225)** | | | |
| --- | --- | --- | --- | --- | --- | --- | --- | --- | --- | --- | --- | --- |
|  | ***B* (*SE*)** | ***p*** | ***OR*** | **95% CI** | ***B* (*SE*)** | ***p*** | ***OR*** | **95% CI** | ***B* (*SE*)** | ***p*** | ***OR*** | **95% CI** |
| **Happy (vs. Neutral)** ^a^ |  |  |  |  |  |  |  |  |  |  |  |  |
| Intercept | -2.28 (0.25) | <.001 |  |  | -2.17 (0.48) | <.001 |  |  | -1.04 (0.32) | .001 |  |  |
| Age | 0.20 (0.10) | .051 | 1.23 | [1.00, 1.50] | 0.15 (0.25) | .532 | 1.17 | [0.72, 1.89] | 0.20 (0.21) | .324 | 1.22 | [0.82, 1.83] |
| Time Spent Online | 0.10 (0.11) | .347 | 1.10 | [0.90, 1.36] | -0.49 (0.26) | .064 | 0.61 | [0.37, 1.03] | 0.14 (0.24) | .565 | 1.15 | [0.72, 1.83] |
| Emotional Problems | -0.09 (0.12) | .437 | 0.91 | [0.72, 1.15] | 0.01 (0.22) | .960 | 1.01 | [0.66, 1.55] | -0.18 (0.19) | .348 | 0.84 | [0.58, 1.21] |
| Sensation Seeking | 0.31 (0.11) | .003 | 1.37 | [1.11, 1.68] | 0.32 (0.19) | .090 | 1.38 | [0.95, 1.99] | 0.50 (0.20) | .012 | 1.66 | [1.12, 2.45] |
| Quality of Family Environment | -0.04 (0.11) | .693 | 0.96 | [0.77, 1.19] | -0.63 (0.20) | .001 | 0.53 | [0.36, 0.78] | -0.06 (0.19) | .740 | 0.94 | [0.65, 1.35] |
| Gender = male | 2.58 (0.28) | <.001 | 13.20 | [7.68, 22.70] | 2.31 (0.56) | <.001 | 10.08 | [3.39, 29.97] | 1.22 (0.41) | .003 | 3.37 | [1.50, 7.56] |
| **Upset (vs. Neutral)** ^a^ |  |  |  |  |  |  |  |  |  |  |  |  |
| Intercept | -0.07 (0.13) | .591 |  |  | 0.15 (0.29) | .605 |  |  | 0.19 (0.24) | .438 |  |  |
| Age | -0.61 (0.09) | <.001 | 0.54 | [0.46, 0.65] | -0.74 (0.24) | .002 | 0.48 | [0.30, 0.76] | -0.76 (0.18) | <.001 | 0.47 | [0.33, 0.66] |
| Time Spent Online | -0.30 (0.10) | .001 | 0.74 | [0.61, 0.89] | -0.49 (0.28) | .080 | 0.61 | [0.35, 1.06] | 0.22 (0.20) | .289 | 1.24 | [0.83, 1.85] |
| Emotional Problems | 0.27 (0.11) | .011 | 1.31 | [1.06, 1.61] | 0.18 (0.20) | .370 | 1.19 | [0.81, 1.76] | 0.12 (0.17) | .490 | 1.13 | [0.81, 1.57] |
| Sensation Seeking | -0.29 (0.11) | .009 | 0.75 | [0.60, 0.93] | -0.39 (0.24) | .097 | 0.68 | [0.42, 1.07] | -0.08 (0.19) | .688 | 0.93 | [0.64, 1.35] |
| Quality of Family Environment | 0.10 (0.10) | .333 | 1.10 | [0.90, 1.35] | -0.17 (0.23) | .453 | 0.84 | [0.54, 1.32] | 0.03 (0.18) | .868 | 1.03 | [0.73, 1.46] |
| Gender = male | -0.40 (0.20) | .047 | 0.67 | [0.45, 0.99] | -2.52 (0.69) | <.001 | 0.08 | [0.02, 0.31] | -0.91 (0.39) | .020 | 0.40 | [0.19, 0.87] |

^a^ response *I felt neither happy nor upset* was used as reference category.

**Supplemental Table 5**: Full Results of Multinomial Logistic Regression (Feelings After SEM Exposure): Poland, Portugal, Romania.

| **Predictors** | **Poland (*N* = 132)** | | | | **Portugal (*N* = 334)** | | | | **Romania (*N* = 177)** | | | |
| --- | --- | --- | --- | --- | --- | --- | --- | --- | --- | --- | --- | --- |
|  | ***B* (*SE*)** | ***p*** | ***OR*** | **95% CI** | ***B* (*SE*)** | ***p*** | ***OR*** | **95% CI** | ***B* (*SE*)** | ***p*** | ***OR*** | **95% CI** |
| **Happy (vs. Neutral)** ^a^ |  |  |  |  |  |  |  |  |  |  |  |  |
| Intercept | -3.66 (1.09) | .001 |  |  | -2.30 (0.39) | <.001 |  |  | -3.82 (1.13) | .001 |  |  |
| Age | -0.45 (0.26) | .082 | 0.64 | [0.39, 1.06] | -0.27 (0.17) | .097 | 0.76 | [0.55, 1.05] | -0.16 (0.24) | .513 | 0.86 | [0.54, 1.37] |
| Time Spent Online | 0.21 (0.28) | .469 | 1.23 | [0.71, 2.14] | 0.37 (0.16) | .022 | 1.45 | [1.06, 2.00] | 0.51 (0.27) | .058 | 1.66 | [0.98, 2.80] |
| Emotional Problems | 0.12 (0.27) | .670 | 1.12 | [0.66, 1.93] | -0.16 (0.18) | .381 | 0.85 | [0.59, 1.22] | 0.46 (0.28) | .098 | 1.58 | [0.92, 2.73] |
| Sensation Seeking | 0.57 (0.26) | .028 | 1.77 | [1.06, 2.96] | 0.44 (0.15) | .003 | 1.54 | [1.16, 2.06] | 0.18 (0.25) | .470 | 1.20 | [0.73, 1.97] |
| Quality of Family Environment | -0.35 (0.24) | .145 | 0.70 | [0.44, 1.13] | -0.26 (0.18) | .149 | 0.78 | [0.55, 1.10] | -0.36 (0.22) | .097 | 0.70 | [0.46, 1.07] |
| Gender = male | 3.27 (1.12) | .004 | 26.41 | [2.93, 238.25] | 2.47 (0.44) | <.001 | 11.86 | [5.02, 28.04] | 3.47 (1.17) | .003 | 32.05 | [3.23, 318.01] |
| **Upset (vs. Neutral)** ^a^ |  |  |  |  |  |  |  |  |  |  |  |  |
| Intercept | -0.03 (0.29) | .924 |  |  | -0.58 (0.21) | .007 |  |  | 0.85 (0.30) | .005 |  |  |
| Age | -0.44 (0.19) | .023 | 0.64 | [0.44, 0.94] | -0.85 (0.18) | <.001 | 0.43 | [0.30, 0.61] | -0.42 (0.18) | .017 | 0.66 | [0.47, 0.93] |
| Time Spent Online | -0.03 (0.22) | .895 | 0.97 | [0.63, 1.49] | -0.13 (0.18) | .463 | 0.88 | [0.62, 1.25] | -0.17 (0.19) | .365 | 0.85 | [0.59, 1.22] |
| Emotional Problems | 0.24 (0.20) | .241 | 1.27 | [0.85, 1.89] | 0.30 (0.19) | .101 | 1.35 | [0.94, 1.95] | 0.25 (0.20) | .213 | 1.28 | [0.87, 1.90] |
| Sensation Seeking | 0.02 (0.23) | .935 | 1.02 | [0.65, 1.60] | -0.23 (0.20) | .248 | 0.80 | [0.54, 1.17] | -0.08 (0.21) | .691 | 0.92 | [0.62, 1.38] |
| Quality of Family Environment | -0.02 (0.18) | .914 | 0.98 | [0.70, 1.38] | -0.25 (0.19) | .179 | 0.78 | [0.54, 1.12] | 0.06 (0.16) | .711 | 1.06 | [0.78, 1.45] |
| Gender = male | -0.38 (0.44) | .394 | 0.69 | [0.29, 1.64] | -0.99 (0.35) | .005 | 0.37 | [0.19, 0.74] | -1.26 (0.43) | .003 | 0.28 | [0.12, 0.66] |

^a^ response *I felt neither happy nor upset* was used as reference category.

**Supplemental Table 6**: Full Results of Multinomial Logistic Regression (Feelings After SEM Exposure): Serbia, Spain, Switzerland.

| **Predictors** | **Serbia (*N* = 386)** | | | | **Spain (*N* = 839)** | | | | **Switzerland (*N* = 259)** | | | |
| --- | --- | --- | --- | --- | --- | --- | --- | --- | --- | --- | --- | --- |
|  | ***B* (*SE*)** | ***p*** | ***OR*** | **95% CI** | ***B* (*SE*)** | ***p*** | ***OR*** | **95% CI** | ***B* (*SE*)** | ***p*** | ***OR*** | **95% CI** |
| **Happy (vs. Neutral)** ^a^ |  |  |  |  |  |  |  |  |  |  |  |  |
| Intercept | -3.81 (0.55) | <.001 |  |  | -0.83 (0.15) | <.001 |  |  | -3.79 (1.12) | .001 |  |  |
| Age | 0.45 (0.17) | .008 | 1.57 | [1.12, 2.20] | -0.12 (0.10) | .239 | 0.89 | [0.73, 1.08] | 0.74 (0.42) | .078 | 2.09 | [0.92, 4.77] |
| Time Spent Online | 0.21 (0.18) | .238 | 1.23 | [0.87, 1.74] | 0.12 (0.09) | .167 | 1.13 | [0.95, 1.34] | -0.10 (0.30) | .741 | 0.91 | [0.50, 1.64] |
| Emotional Problems | -0.04 (0.21) | .864 | 0.97 | [0.64, 1.45] | -0.13 (0.10) | .178 | 0.88 | [0.73, 1.06] | 0.29 (0.29) | .314 | 1.34 | [0.76, 2.37] |
| Sensation Seeking | 0.36 (0.15) | .019 | 1.43 | [1.06, 1.94] | 0.17 (0.08) | .045 | 1.18 | [1.00, 1.39] | 0.53 (0.27) | .044 | 1.70 | [1.01, 2.86] |
| Quality of Family Environment | 0.27 (0.21) | .196 | 1.31 | [0.87, 1.98] | -0.12 (0.09) | .146 | 0.88 | [0.75, 1.04] | -0.46 (0.23) | .041 | 0.63 | [0.41, 0.98] |
| Gender = male | 3.80 (0.58) | <.001 | 44.65 | [14.46, 137.94] | 1.38 (0.19) | <.001 | 3.97 | [2.75, 5.73] | 3.30 (1.16) | .004 | 27.06 | [2.81, 260.74] |
| **Upset (vs. Neutral)** ^a^ |  |  |  |  |  |  |  |  |  |  |  |  |
| Intercept | -0.23 (0.19) | .229 |  |  | -0.65 (0.15) | <.001 |  |  | 1.36 (0.26) | <.001 |  |  |
| Age | -0.69 (0.16) | <.001 | 0.50 | [0.37, 0.68] | -0.69 (0.11) | <.001 | 0.50 | [0.41, 0.62] | -0.63 (0.23) | .007 | 0.53 | [0.34, 0.84] |
| Time Spent Online | -0.22 (0.16) | .163 | 0.80 | [0.58, 1.10] | 0.01 (0.10) | .944 | 1.01 | [0.83, 1.22] | -0.30 (0.22) | .176 | 0.74 | [0.48, 1.14] |
| Emotional Problems | 0.35 (0.15) | .020 | 1.42 | [1.06, 1.90] | 0.18 (0.11) | .097 | 1.19 | [0.97, 1.47] | 0.25 (0.20) | .210 | 1.28 | [0.87, 1.89] |
| Sensation Seeking | -0.37 (0.17) | .025 | 0.69 | [0.50, 0.96] | -0.21 (0.11) | .045 | 0.81 | [0.66, 1.00] | -0.34 (0.22) | .117 | 0.71 | [0.46, 1.09] |
| Quality of Family Environment | 0.31 (0.17) | .060 | 1.37 | [0.99, 1.89] | -0.04 (0.10) | .711 | 0.97 | [0.80, 1.17] | 0.33 (0.18) | .071 | 1.39 | [0.97, 2.00] |
| Gender = male | -1.46 (0.37) | <.001 | 0.23 | [0.11, 0.48] | -0.20 (0.20) | .318 | 0.82 | [0.56, 1.21] | -1.68 (0.36) | <.001 | 0.19 | [0.09, 0.38] |

^a^ response *I felt neither happy nor upset* was used as reference category.

**Supplemental Table 7**: Likelihood Ratio Tests for Predictors in Multinomial Logistic Regression (Feelings After SEM Exposure): Czechia, Finland, Malta.

| **Predictors** | **Czechia** | | | **Finland** | | | **Malta** | | |
| --- | --- | --- | --- | --- | --- | --- | --- | --- | --- |
|  | **χ²** | **df** | ***p*** | **χ²** | **df** | ***p*** | **χ²** | **df** | ***p*** |
| Age | 43.87–74.22 | 2 | <.001 | 11.15–14.38 | 2 | .001–.004 | 18.47–31.73 | 2 | <.001 |
| Time Spent Online | 13.46–18.21 | 2 | <.001–.001 | 4.62–9.22 | 2 | .010–.099 | 0.22–2.22 | 2 | .329–.842 |
| Emotional Problems | 7.64–10.86 | 2 | .004–.022 | 0.34–2.59 | 2 | .274–.843 | 1.28–3.80 | 2 | .150–.527 |
| Sensation Seeking | 17.43–29.06 | 2 | <.001 | 3.98–10.28 | 2 | .006–.137 | 8.10–12.80 | 2 | .002–.017 |
| Quality of Family Environment | 0.40–2.96 | 2 | .228–.691 | 9.92–16.95 | 2 | <.001–.007 | 0.01–1.88 | 2 | .391–.995 |
| Gender = male | 150.07–159.39 | 2 | <.001 | 49.20–66.01 | 2 | <.001 | 20.88–27.95 | 2 | <.001 |

*Note.* We present ranges of χ² statistics and *p*-values across the 20 imputations.

**Supplemental Table 8**: Likelihood Ratio Tests for Predictors in Multinomial Logistic Regression (Feelings After SEM Exposure): Poland, Portugal, Romania.

| **Predictors** | **Poland** | | | **Portugal** | | | **Romania** | | |
| --- | --- | --- | --- | --- | --- | --- | --- | --- | --- |
|  | **χ²** | **df** | ***p*** | **χ²** | **df** | ***p*** | **χ²** | **df** | ***p*** |
| Age | 5.72–7.35 | 2 | .025–.053 | 21.92–25.82 | 2 | <.001 | 5.40–8.52 | 2 | .031–.067 |
| Time Spent Online | 0.27–3.10 | 2 | .401–.875 | 5.17–9.25 | 2 | .010–.075 | 5.85–7.48 | 2 | .024–.054 |
| Emotional Problems | 0.60–3.15 | 2 | .317–.740 | 3.24–5.45 | 2 | .065–.198 | 2.22–4.78 | 2 | .092–.330 |
| Sensation Seeking | 3.85–8.46 | 2 | .015–.124 | 9.38–14.42 | 2 | .001–.004 | 0.27–2.24 | 2 | .326–.876 |
| Quality of Family Environment | 1.29–4.33 | 2 | .134–.525 | 2.57–4.45 | 2 | .108–.277 | 2.47–5.43 | 2 | .066–.290 |
| Gender = male | 18.51–23.17 | 2 | <.001 | 57.37–73.47 | 2 | <.001 | 36.56–42.77 | 2 | <.001 |

*Note.* We present ranges of χ² statistics and *p*-values across the 20 imputations.

**Supplemental Table 9**: Likelihood Ratio Tests for Predictors in Multinomial Logistic Regression (Feelings After SEM Exposure): Serbia, Spain, Switzerland.

| **Predictors** | **Serbia** | | | **Spain** | | | **Switzerland** | | |
| --- | --- | --- | --- | --- | --- | --- | --- | --- | --- |
|  | **χ²** | **df** | ***p*** | **χ²** | **df** | ***p*** | **χ²** | **df** | ***p*** |
| Age | 33.40–38.63 | 2 | <.001 | 43.69–48.88 | 2 | <.001 | 14.90–16.57 | 2 | <.001–.001 |
| Time Spent Online | 2.67–6.79 | 2 | .034–.263 | 1.44–3.58 | 2 | .167–.487 | 1.37–2.66 | 2 | .264–.505 |
| Emotional Problems | 5.31–6.50 | 2 | .041–.070 | 6.12–9.62 | 2 | .008–.047 | 1.25–3.66 | 2 | .160–.536 |
| Sensation Seeking | 9.95–15.90 | 2 | <.001–.004 | 11.35–17.94 | 2 | <.001–.003 | 7.25–12.98 | 2 | .002–.027 |
| Quality of Family Environment | 4.23–6.27 | 2 | .043–.120 | 1.18–3.20 | 2 | .202–.554 | 8.04–14.78 | 2 | .001–.017 |
| Gender = male | 124.05–133.05 | 2 | <.001 | 73.70–81.62 | 2 | <.001 | 48.56–61.98 | 2 | <.001 |

*Note.* We present ranges of χ² statistics and *p*-values across the 20 imputations.
